# A non-traditional endoscopic approach to laryngeal schwannoma

**DOI:** 10.1016/j.bjorl.2024.101400

**Published:** 2024-02-22

**Authors:** Petru Gurău

**Affiliations:** “Timofei Moșneaga” Republican Clinical Hospital, Department of Thoracic Surgery, Chișinău, Republic of Moldova

## Introduction

Laryngeal schwannomas are rare benign nerve sheath tumors that represent up to 1.5% of all benign laryngeal tumors.^1^ All laryngeal schwannomas are encapsulated submucosal tumors that arise predominantly in the supraglottis^1^ and originate mostly from the internal branch of the superior laryngeal nerve.^1^, ^2^ The symptoms usually develop over years and include hoarseness/dysphonia, inspiratory dyspnea, and foreign body sensation during swallowing.^2^ The diagnosis is based, mainly, on flexible laryngoscopy, imaging techniques, and histological exam.

A case of non-typically located laryngeal schwannoma is reported below, and a non-traditional endoscopic approach to treating obstructive laryngeal schwannoma as a means of avoidance of external surgical approach and minimizing of surgical trauma is discussed.

## Case report

A 29-year-old man presented with a 12-year history of progressive hoarseness and inspiratory dyspnea. Six years ago, the patient was consulted in another hospital, a larynx tumor was detected, and open laryngeal surgery was proposed, but the patient rejected the operation.

**Figure 1:**
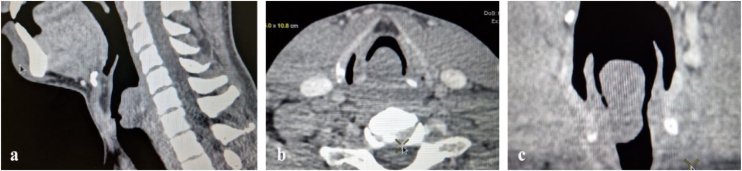
Computed tomography image of laryngeal schwannoma. (a) Sagittal view; (b) axial view; (c) coronal view.

Computed tomography examination with contrast showed a broad-based hypodense mass, emerging from the posterior and right lateral wall of the larynx, with dimensions of 3.6 × 1.8 × 1.6 cm, occupying all three parts of the larynx and obstructing approximately 80% of the laryngeal lumen, without signs of erosion of the adjacent cricoid cartilage (Fig. 1).

**Figure 2:**
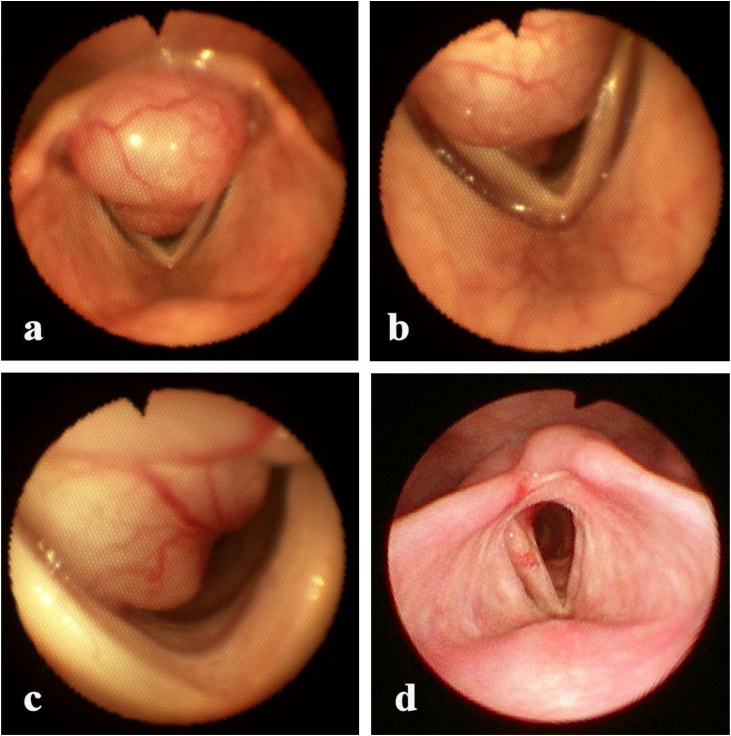
Endoscopic appearance of laryngeal schwannoma. (a) Supraglottic portion; (b) glottic portion; (c) subglottic portion; (d) 7-months after surgery: no visible laryngeal tumor.

Flexible laryngoscopy revealed on the posterior laryngeal wall an exophytic broad-based tumor, with irregular shape and hard-elastic consistency, the surface being smooth and glossy with accentuated vascular pattern, with approximate dimensions of 4.0 × 2.0 × 2.0 cm, that considerably obstructed the lumen of supraglottic, glottic and subglottic parts of the larynx. The superior margin of the tumor was appreciated 0.3 cm above the upper margin of the arytenoids, and the inferior margin of the tumor was appreciated 1.5 cm below the vocal cords. The endoscopic appearance was suggestive of a benign non-epithelial laryngeal tumor (Fig. 2a‒c).

Endoscopic management was selected as the first-line approach for this case. Written informed consent was obtained from the patient. After preventive tracheostomy, direct suspension rigid laryngoscopy combined with flexible laryngoscopy using a therapeutic flexible bronchoscope under superimposed high-frequency jet ventilation was performed. Initially, incisions using a 980/1470 nm diode laser (20 W) were made at the upper and lower (via tracheotomy orifice) margins of the tumor base, the flexible laser guide being introduced through the working channel of the flexible bronchoscope. In the following step, cold resection/coring out of the tumor by the bevel of the rigid bronchoscope was performed. A minor bleeding/oozing was encountered after cold resection. Finally, laser coagulation of the bleeding surface and vaporization of tumor remnants was performed at the base and margins of the tumor bed. The patient was decannulated 3 days later and discharged home on the fifth postoperative day. The histological exam revealed a schwannoma.

Flexible laryngoscopy, performed 7-months after the operation, showed no tumor recurrence, free laryngeal lumen, and minor scar changes of the mucosa of the posterior wall of the larynx (Fig. 2d). The patient’s voice and respiration were completely restored.

## Discussion

The larynx is an extremely rare location for schwannomas. Approximately 130 cases had been reported in the literature up to 1993.^2^

The most common laryngeal schwannoma is located in the false vocal folds (45.8%), followed by the aryepiglottic folds (33.3%), less frequently it arises from the true vocal folds (16.7%), epiglottis (9.7%), subglottis (5.6%) and postcricoid area (4.1%).^3^ Typical finding on laryngoscopy is a round submucosal bulge in the region of the false vocal fold or aryepiglottic fold.^1^

Surgery is the mainstay for the treatment of laryngeal schwannomas. Transoral surgery is recommended for small and pedunculated lesions and an external approach (lateral pharyngotomy, lateral thyrotomy, or laryngofissure) is recommended for large obstructive tumors.^1^, ^3^ The prognosis after complete surgical excision of the tumor is, generally, good. Recurrent/persistent lesions after surgery are encountered in about 17% of non-pedunculated tumors with no statistically significant difference between endoscopic and open procedures.^3^

There is no consensus about follow-up after surgery for laryngeal schwannoma. Tulli et al. mention in the most comprehensive review on laryngeal schwannoma that recurrent disease in almost all patients was identified within 3-months of surgery and suggest performing flexible laryngoscopy every 3-months for the first year and then annually for at least 2-years after surgery.^3^

Concerning the presented case, a strong desire of the patient to avoid open laryngeal surgery was primarily taken into consideration (six years before the patient rejected an open surgery proposal). The surgeon who performed the intervention has experience in interventional bronchoscopy and applied some techniques used in interventional bronchoscopy for the described case. It is not uncommon to treat endotracheal and endobronchial tumors using fiber-based lasers and to resect them by coring using a rigid bronchoscope,^4^ but we have not found in the literature any descriptions of using such a technique in endolaryngeal surgery.

Summarizing the particularities of the presented case, we would like to mention the following reasons that make it, in our opinion, a special one:

• Tumor location was not typical: the broad-based tumor was arising from the posterior wall of the larynx (on the cricoid cartilage and interarytenoid fold);
• Tumor extension was not typical: the tumor occupied all three parts of the larynx: supraglottis, glottis, and subglottis;
• Tumor dimensions were not typical: approximately, 4.0 cm in its largest dimension;
• The therapeutic approach was not typical: we have not found in the accessible literature a description of an endoscopic approach to the broad-based tumor with such dimensions affecting subglottis (normally, an external approach is chosen in such cases);
• The tools that were used for resolving this case were not typical: flexible bronchoscope, diode laser, and rigid bronchoscope.

## Conclusion

The presented case demonstrates that even big obstructive laryngeal schwannomas, that affect all three parts of the larynx, can be successfully eradicated by endoscopic surgery in selected cases, combining such tools as flexible bronchoscope, laser, and rigid bronchoscope, provided the absence of extraluminal growth of the tumor. The described technique could add to the diversity of approaches in the management of this rare entity.

## Funding

This research did not receive any specific grant from funding agencies in the public, commercial, or not-for-profit sectors.

## Consent statement

Informed consent was obtained from the patient for the publication of this case report.

## Ethics approval statement

This manuscript was approved by the “Timofei Moșneaga” Republican Clinical Hospital Institutional Ethics Committee. The research was conducted ethically, with all study procedures performed in accordance with the requirements of the World Medical Association’s Declaration of Helsinki. Patient’s data are not identifiable. The patient did not receive any stipend for participation in the study.

## Data and materials availability statement

All data generated or analyzed during this study are included in this article. Further enquiries can be directed to the corresponding author.

## Conflicts of interest

The author declares no conflicts of interest.
